# Analysis of human meiotic recombination events with a parent-sibling tracing approach

**DOI:** 10.1186/1471-2164-12-434

**Published:** 2011-08-26

**Authors:** Yun-Shien Lee, Angel Chao, Chun-Houh Chen, Tina Chou, Shih-Yee Mimi Wang, Tzu-Hao Wang

**Affiliations:** 1 Department of Biotechnology, Ming Chuan University, Tao-Yuan, Taiwan; 2 Genomic Medicine Research Core Laboratory (GMRCL), Chang Gung Memorial Hospital, Tao-Yuan, Taiwan; 3 Department of Obstetrics and Gynecology, Lin-Kou Medical Center, Chang Gung Memorial Hospital and Chang Gung University, Tao-Yuan, Taiwan; 4 Institute of Statistical Science, Academia Sinica, Taipei, Taiwan; 5 Department of Obstetrics and Gynecology, White Memorial Medical Center, Los Angeles, CA, USA

## Abstract

**Background:**

Meiotic recombination ensures that each child inherits distinct genetic materials from each parent, but the distribution of crossovers along meiotic chromosomes remains difficult to identify. In this study, we developed a parent-sibling tracing (PST) approach from previously reported methods to identify meiotic crossover sites of GEO GSE6754 data set. This approach requires only the single nucleotide polymorphism (SNP) data of the pedigrees of both parents and at least two of children.

**Results:**

Compared to other SNP-based algorithms (identity by descent or pediSNP), fewer uninformative SNPs were derived with the use of PST. Analysis of a GEO GSE6754 data set containing 2,145 maternal and paternal meiotic events revealed that the pattern and distribution of paternal and maternal recombination sites vary along the chromosomes. Lower crossover rates near the centromeres were more prominent in males than in females. Based on analysis of repetitive sequences, we also showed that recombination hotspots are positively correlated with SINE/MIR repetitive elements and negatively correlated with LINE/L1 elements. The number of meiotic recombination events was positively correlated with the number of shorter tandem repeat sequences.

**Conclusions:**

The advantages of the PST approach include the ability to use only two-generation pedigrees with two siblings and the ability to perform gender-specific analyses of repetitive elements and tandem repeat sequences while including fewer uninformative SNP regions in the results.

## Background

Meiotic recombination is important for generating genetic diversity. Meiotic recombination occurs between homologous chromosomes during chiasmata formation, a process that is required for normal chromosomal segregation during meiosis. While variation in recombination rates is a ubiquitous feature of the human genome [1], the mechanisms governing the distribution of crossovers along meiotic chromosomes remain largely unclear, with the exception of the recent discovery that Prdm9 is involved in the activation of mammalian recombination hotspots [2-5]. Sex-specific effects [6-8] on regional meiotic recombination have been described. Recombination rates are approximately 1.7-fold higher in female meiosis than in male meiosis. In addition, crossover rates in males are 5-fold lower near centromeres but 10-fold higher near telomeres compared with those in females [9]. These differences could be related to sex-specific patterns of initiation of synapses between homologs. For example, synaptonemal complex lengths are shorter in males than in females [10], and synapses appear preferentially in subtelomeric regions in males [11].

Meiotic recombination events can be measured directly or indirectly [12]. Physical crossovers between homologous chromosomes, indicating meiotic recombination events, can be directly observed at specific time points during spermatogenesis [13]. Alternatively, crossovers may be analyzed directly in cytogenetic analysis by labeling meiosis-related proteins, such as MLH1 [14]. Despite the unequivocal value of direct analysis, these techniques are labor-intensive and precision is limited. Therefore, most analyses of human recombination currently rely on indirect approaches such as genetic linkage analysis of human pedigrees. This involves tracking the inheritance of alleles at multiple polymorphic markers (short tandem repeat polymorphisms, STRP; or single nucleotide polymorphisms, SNP) along the chromosomes across generations [15-17].

**Figure 1:**
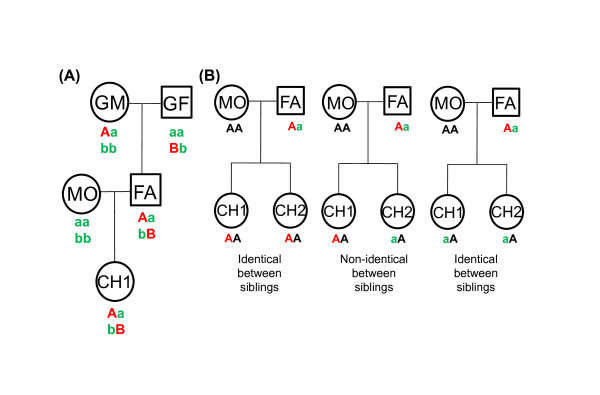
**Different types of pedigrees are required for determining meiotic recombination sites by various methods**. **(A) **Three-generation pedigrees are required for the identity by descent (IBD) method, and **(B) **complete two-generation pedigree for the parent-sibling tracing (PST) method. In the IBD method, the 'A' and 'B' allele in child 1 were required to originate from grandmother and grandfather, respectively. In PST approach, the paternal genotype was 'Aa' and the maternal genotype was 'AA', children with 'Aa' and 'aa' were coded as "0: not identical between siblings". If both children were 'Aa' and 'Aa' [or ('AA' and 'AA')], they were coded as "1: identical between siblings", (identical genotype origin for both children). ***Abbreviations***: GF, grandfather; GM, grandmother; FA, father; MO, mother; CH1 and CH2, child 1 and child 2.

Molecular markers in individuals with known pedigrees can be traced to an ancestral identity using either the identity by descent (IBD) method [12] or the identity by state (IBS) method [18]. Two alleles at a particular locus in the progeny are assumed to be identical if they are derived from an identical locus in a common ancestor. The IBD method requires knowledge of the genotypes of three generations to determine if the DNA segments are identical by descent from each generation. In the IBD method, shared results between each child and his/her paternal and maternal grandparents are analyzed separately. A paternal recombination event is detected when the IBD sharing "switches" from one paternal grandparent to the other. This application can be applied in the same manner for the maternal side. For instance, meiotic events can be switched between 2 SNP sites (Figure 1A and Additional File 1A). Therefore, application of the IBD method requires the pedigrees of three generations [12]. The IBS method was used to detect meiotic recombination sites between individuals by analyzing allele sharing between siblings [18]. Recently, Ting et al. also proposed another method for identifying meiotic recombination patterns based on two-generation pedigrees (pediSNP) [19]. In the pediSNP method, genotypes of two children are analyzed and compared with the genotype of one parent [19].

Based on the distribution of SNPs in both parents and multiple siblings, meiotic cross sites in human chromosomes can be identified. This method was first proposed by Coop et al. in 2008 to trace the "informative markers" transmitted by the father to each offspring [6]. They defined the "informative markers" as SNPs that are heterozygous in the father and homozygous in the mother. In 2009, Chowdhury et al. used two datasets, namely, the Autism Genetic Research Exchange (AGRE) and the Framingham Heart Study (FHS), to characterize the variation in recombination phenotypes [20]. They analyzed sex differences and recombination jungles across the human genome, and described the gene loci associated with recombination phenotypes [20].

In this study, we have used a parent-sibling tracing (PST) approach, which was derived from two previous reports [6,20], to analyze the Genomic Medicine Research Core Laboratory, Taiwan (GMRCL) dataset of Affymetrix SNP6.0 arrays which consists of 900 K SNP markers and the GSE6754 dataset from Gene Expression Omnibus (GEO) [21], which consists of 853 families. Our analyses of this dataset of 2,145 meioses resulted in a 1-Mb-resolution recombination map. In addition, we were able to characterize the relationships between recombination sites and repetitive elements as well as the relationships between recombination sites and tandem repeats sequences.

## Results

### Comparison of two methods of detecting meiotic recombination sites

**Figure 2:**
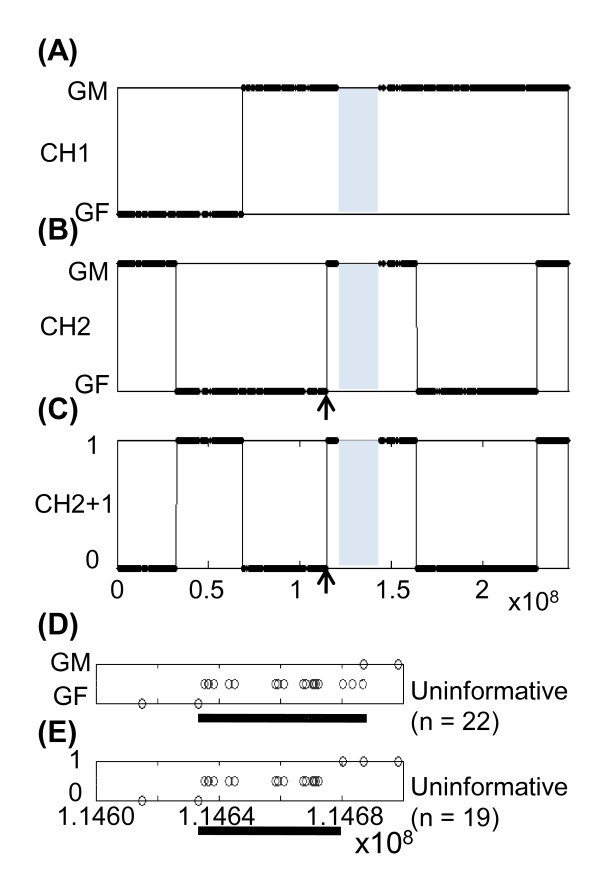
**The paternal recombination site on chromosome 1 of child 1 and 2 (CH1 and CH2, defined in Figure 1) in the GMRCL dataset were defined using the identity by descent (IBD) (**A, B, D**) and parent-sibling tracing (PST) (**C, E**) methods**. The grandmother and grandfather origin of paternal recombination is indicated as GM and GF, respectively. Children with identical or not identical origin are indicated as 1 and 0, respectively. Panels **D **and **E **are the enlarged view of the 114.6 -114.7 kb region on chromosome 1 shown in panels **B **and **C**, respectively, which are indicated by the black arrows. **D **and **E**: the SNP sites (open circles) that could not be mapped to either GF or GM in the IBD method, or to either an identical or non-identical status using the PST approach, are indicated as a uninformative SNPs. The calling schema of IBD and PST methods is shown in Additional File 1. The chromosomal regions without any SNP site in the Affymetrix Genome-Wide Human SNP 6.0 arrays are marked as gray blocks (**A **to **C**).

We used the GMRCL dataset of 900 K SNPs as a reference standard for comparison between the PST approach (Figure 1B) and previous approaches such as the IBD method [12] (Figure 1A). The code calling schema of PST is depicted in Figure 1B and Additional File 1B. Using chromosome 1 as an example, IBD analysis in both children could define the sites of meiotic recombination for paternal gametes. In child 1 and child 2, we observed 1 and 4 meiotic recombination events on their paternal gametes, respectively (Figures 2A and 2B). Using the PST approach, we could analyze the paternal genotypes for both children. When the paternal genotype was Aa and the maternal genotype was AA, children with Aa and AA were coded as "0: not identical between siblings". If both children were Aa and Aa [or (AA and AA)], they were coded as "1: identical between siblings" (identical genotype origin for both children). The PST approach (Figure 2C) detected the recombination sites of the combinatorial results for child 1 and child 2 as determined by IBD (Figures 2A and 2B). These results indicate that, using the SNP information of only two generations, PST can identify the origin of the recombination site. For the IBD method, information from three generations is required to determine whether the origin is from the grandfather or the grandmother. The 43 recombination sites identified in the GMRCL dataset using the IBD and PST methods are shown in Additional file 2.

Comparison of the code calling schemas between the IBD and PST methods showed that IBD identified fewer genotyping combination calls than the PST approach. For instance, when we analyzed the recombination sites in the 100-kb genomic region located at 114.6 Mb on chromosome 1 (Figures 2B and 2C, indicated with the arrow), the numbers of uninformative SNPs in the recombination site for the IBD and PST methods were 22 and 19, respectively (Figures 2D and 2E), resulting in uninformative regions of 54 kb for the IBD method (Figure 2D) and 48 kb for the PST approach (Figure 2E), respectively.

The use of the IBD and PST methods in the GMRCL sample led to the identification of 43 paternal recombination sites in child 1 and child 2. The mean numbers of uninformative SNP for the 43 paternal recombination sites were 71.2 and 36.7 for the IBD and PST methods, respectively (Table 1). The mean sizes of the uninformative regions for the 43 paternal recombination sites were 253 ± 349 kb (mean ± SD) with 110 (58 - 336) in Q2 (Q1-Q3) for the IBD method, and 167 ± 391 kb with 60 (23 - 157) in Q2 (Q1-Q3) for the PST approach (Table 1). The paired t-test showed that the PST approach resulted in significantly shorter uninformative regions than the IBD method (P < 10^-10^).

**Table 1:** Comparison of the size and SNP numbers in uninformative regions

		IBD		PST	
	**Sibling#**	**Q2 (Q1 - Q3) kb **^†^	SNP#*	**Q2 (Q1 - Q3) kb **^†^	SNP#*

*900 K*					
**Paternal**	2	110 (58 - 336)	**71.2**	60 (23 - 157)	**36.7**
**Maternal**	2			61 (19 - 189)	**39.6**

*Autism_3117*					
**Paternal**	4	3291 (2255 - 5738)	**12.31**	1751 (1270 - 3347)	**7.1**
**Maternal**	4	2683 (1249 - 5796)	**15.34**	1806 (947 - 3389)	**5.9**

*Autism_3180*					
**Paternal**	6	3768 (1858 - 6420)	**16.1**	1701 (938 - 2853)	**5.8**
**Maternal**	6	2842 (1145 - 5789)	**14.6**	2151 (1234 - 3712)	**6.9**

*Autism_8071*					
**Paternal**	4	3557 (1877 - 6415)	**15.6**	1892 (1195 - 3230)	**7.5**
**Maternal**	4			2046 (1130 - 4031)	**7.8**

† Q2 (Q1 - Q3): Q2 (second quartile) = 50th percentile; Q1 (first quartile) = 25th percentile; Q3 (third quartile) = 75th percentile

* SNP#: Average number of SNPs in the "uninformative" region

### Analysis of the GEO dataset GSE6754 containing 11,000 SNP markers

The Affymetrix Human Mapping 10 K 2.0 Arrays (containing 10 K SNPs) were used to map autism susceptibility loci in the GSE6754 dataset [22]. Three three-generation pedigrees (family ID: 3117, 3180, 8071) were selected to compare the usefulness of the IBD and PST methods. Since the 10 K 2.0 array covered fewer SNPs, the mean size of uninformative regions were about 20-fold higher and the number of uninformative SNPs was approximately 6-fold lower than those of SNP 6.0 Arrays. Compared to other approaches, the PST approach identified fewer uninformative SNPs and smaller uninformative genomic regions (Table 1).

**Figure 3:**
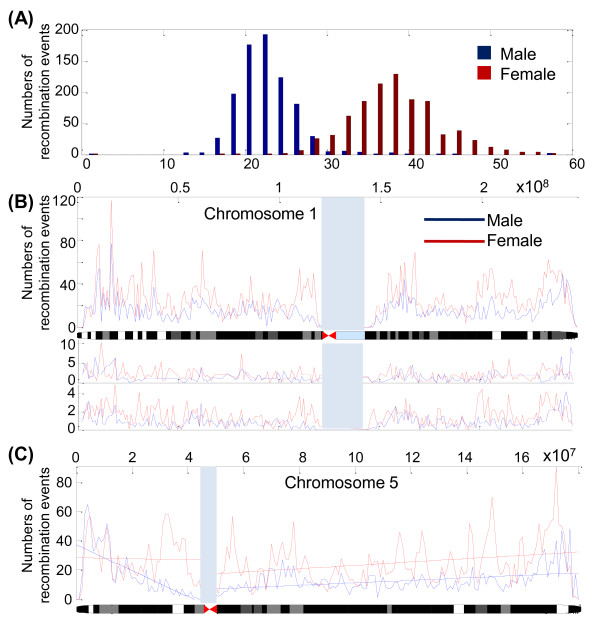
**Distribution of the 2,145 paternal and 2,145 maternal recombination events across all human autosomal chromosomes (A), chromosome 1 (B) and chromosome 6 (C)**. (**A**) The distribution of the numbers of the paternal (blue bar) and maternal (red bar) recombination events across autosomal chromosomes. (**B**) The number of recombination sites for chromosome 1 was calculated using a window width of 1 Mb. The middle and lower panel of the Figure 3B are the Marshfield recombination map and Icelandic recombination map, respectively. The maternal (red) and paternal (blue) genetic distance for each 1-Mb window was calculated on the basis of the SNP position information provided by Affymetrix. We assumed a constant crossover rate between two adjacent SNP markers. The physical position and the chromosome ideogram are shown on the top and bottom of the figure, respectively. (**C**) The regression lines for maternal (red) and paternal (blue) crossover rates corresponding to the distance from the centromere are shown, using chromosome 6 as an example. The slope was significantly different from zero in the p arm of male but not in female chromosomes. In contrast, both genders showed a significant correlation in the number of recombination sites towards the telomere of the q arm. The chromosomal regions without any SNP site in the Affymetrix Genome-Wide Human SNP 6.0 arrays are marked as gray blocks.

In the 3864 arrays (853 families, 1721 parents, 2145 siblings) analyzed using the PST approach, the mean number of maternal recombination events was approximately 1.67-fold higher than that of paternal origin, with the highest value observed on chromosome 17 (2.00-fold) and the lowest on chromosome 22 (1.32-fold) (Table 2). The distribution of recombination events of paternal origin (mean 23.8 ± 4.1, median 22.5) and maternal origin (mean 39.5 ± 5.7, median 38.0) is presented in Figure 3A. The numbers of recombination events of each chromosome (2,145 maternal and paternal meioses) are summarized in Table 2.

**Table 2:** Number of recombination sites in 2145 siblings from 853 families

Chromosome	Male	Female	Female/male
chr1	3990	6819	1.71
chr2	3917	6723	1.72
chr3	3507	5847	1.67
chr4	3007	5361	1.78
chr5	2864	5255	1.83*
chr6	2971	5063	1.70
chr7	2582	4560	1.77
chr8	2378	4212	1.77
chr9	2495	3883	1.56
chr10	2544	4417	1.74
chr11	2348	4017	1.71
chr12	2503	4140	1.65
chr13	1996	3162	1.58
chr14	2007	2784	1.39*
chr15	1988	2859	1.44*
chr16	1762	2902	1.65
chr17	1393	2783	2.00*
chr18	1856	2903	1.56
chr19	1210	2072	1.71
chr20	1612	2383	1.48*
chr21	1004	1444	1.44*
chr22	965	1265	1.31*
chrX		2932	

* P value < 0.01 (chi square analysis under the null hypothesis that the male-to-female proportion was 1.667)

In order to identify the regions with the highest and the lowest number of recombination events, we scanned the entire human genome. We first divided the genome into 2,765 bins of 1-Mb each. We then identified the number of recombination sites in each bin separately for female and male meioses. The results obtained from chromosome 1 are shown in Figure 3B (see the Additional file 3 for the results on other chromosomes). We also compared the recombination maps obtained from dataset GSE6754 with Marshfield map [23] (Figure 3B, middle panel), and Icelandic map [16] (Figure 3B, lower panel). The correlation coefficients between the data in GSE6754 map and Icelandic map and Marshfield map were r = 0.49 and r = 0.31, respectively.

To test the hypothesis that recombination rates are lower near the centromere but higher near the telomeres in men, we analyzed the correlation between the distances from the recombination sites to the centromere and the number of recombination sites. We found significant correlations (P < 0.00001) on chromosomes 1q, 2p, 3q, 4q, 5p, 5q, 6p, 6q, 7q, 8q, 9p, 9q, 10p, 10q, 11q, 12p, 12q, 16q, 18q, 19q, 20q, 21q in men. In contrast, similar correlations were found only on chromosome 1q and 6q in women (Table 3). For instance, the slope of correlation was significant in p arm of chromosome 5 in men but not in women (Figure 3C). On the other hand, both sexes showed significant correlations in the number of recombination sites near the telomere in the q arm. SNP information was not available for the p arm of chromosomes 13, 14, 15, 21, and 22.

**Table 3:** Correlation of the distance from the recombination site to the centromere with the number of recombination events

	Male		Female	
**Chromosome**	**p arm**	**q arm**	**p arm**	**q arm**

chr1	0.03979	**4.1E-10**	0.63413	**3.7E-10**
chr2	**3.1E-07**	0.12	0.00783	0.01009
chr3	0.00127	**1.1E-11**	0.00022	3.9E-05
chr4	0.00865	**2.1E-09**	0.20608	0.00063
chr5	**1.9E-07**	**5.3E-06**	0.87512	0.00262
chr6	**3.4E-07**	**6.7E-16**	0.10238	**5.7E-08**
chr7	0.00329	**2.2E-07**	0.9658	0.00189
chr8	3.9E-05	**2.6E-06**	0.33193	0.41184
chr9	**2.3E-12**	**1.9E-08**	0.00064	0.01546
chr10	**2.3E-09**	**1.6E-07**	0.92443	0.00077
chr11	0.01294	**1.1E-08**	0.83831	4E-05
chr12	**6.2E-06**	**3.6E-06**	0.17744	0.00675
chr13	NA	0.88298	NA	0.41116
chr14	NA	0.42025	NA	0.0348
chr15	NA	0.10395	NA	0.00605
chr16	0.09796	**6.8E-09**	0.88747	0.34803
chr17	0.7478	0.00062	0.15536	0.69596
chr18	0.02079	**2E-09**	0.3442	0.22101
chr19	0.95524	**4.7E-10**	0.06423	0.11279
chr20	4.1E-05	**2.6E-07**	0.33546	0.04724
chr21	NA	**2.3E-10**	NA	0.66508
chr22	NA	0.0005	NA	0.05598

*NA: SNP information not available for the p arm of chromosomes 13, 14, 15, 21, and 22.

The values in bold indicate a P value < 1E5

### Relation between the recombination site and repetitive elements

**Figure 4:**
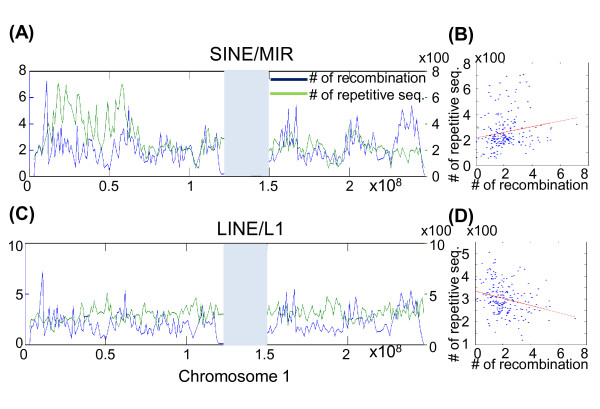
**Correlation between the number of sex-averaged recombination sites and SINE/MIR (A, B) or LINE/L1 (C, D) repetitive sequences elements**. The distribution of the number of sex-averaged recombination sites (blue) and repetitive sequences elements (green) on chromosome 1 was calculated using a window width set to 1 Mb (**A, C**). The scatter plot shows the number of sex-averaged recombination sites and repetitive sequences on chromosome 1 (**B, D**). Regression lines are marked in red. The chromosomal regions without any SNP site in the Affymetrix Genome-Wide Human SNP 6.0 arrays are marked as gray blocks.

We compiled 57 major repetitive element classes that were characterized by RepeatMasker [24]. Twenty-three repetitive-element classes were identified in more than 6,000 sites in the human genome. After downloading the location information of the human CpG islands from the UCSC database [25], we divided the genome into 2,765 bins of 1-Mb each and determined the number of repetitive-element sites in each bin. Using the 53,487 repetitive-elements on chromosome 1 as an example, we depicted the distribution of SINE/MIR (green lines in Figure 4A) and LINE/L1 sites (green lines in Figure 4C). In addition, the distributions of meiotic recombination sites (both paternal and maternal combined) are shown as blue lines. In each 1-Mb bin, we also analyzed the correlation between the number of meiotic recombination sites and the number of SINE/MIR (plotted in Figure 4B) and LINE/L1 sites (plotted in Figure 4D). The correlation coefficients between recombination sites and SINE/MIR and the correlation coefficients between recombination sites and LINE/L1 were 0.23 (P = 0.0005) and 0.29 (P = 0.00001), respectively.

**Figure 5:**
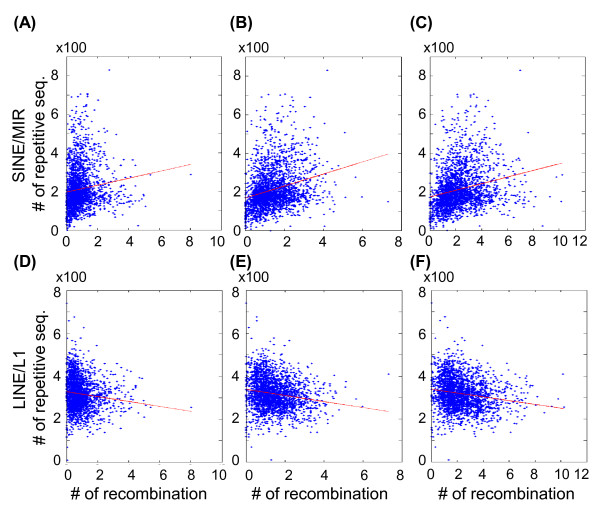
**Scatter plot of the number of paternal (A, D), maternal (B, E), and sex-averaged (C, F) recombination sites for the SINE/MIR (A, B, C) and LINE/L1 (D, E, F) repetitive sequences on chromosome 1**. Regression lines are marked in red.

The correlation coefficients and the corresponding P values for each of the 23 repetitive-elements, CpG island sites, and meiotic recombination sites are summarized in Table 4. The repetitive elements SINE/MIR, DNA/hAT-Charlie, DNA/hAT, LINE/L2, SINE/Alu, DNA/hAT-Tip100, DNA/hAT-Blackjack were positively correlated with meiotic recombination sites. In contrast, repetitive elements, which included LINE/L1, LTR/ERVK, and Low complexity (Table 4), showed negative correlation with meiotic recombination sites. In general, we found no significant differences in the distribution of maternal and paternal recombination sites. The scatter plots of the correlation analyses of repetitive elements SINE/MIR and LINE/L1 in the entire human genome are shown in Figure 5.

**Table 4:** Correlation between the recombination sites and particular repeats

		Paternal		Maternal		Both	
**Repeat**	**Number**	**Corr**	**P**	**Corr**.	**P**	**Corr**.	**P**

SINE/MIR	510580	0.22	**1E-16**	0.30	**1E-16**	0.29	**1E-16**
DNA/hAT-Charlie	214901	0.18	**1E-16**	0.31	**1E-16**	0.29	**1E-16**
DNA/hAT	10624	0.16	**3E-13**	0.24	**1E-16**	0.23	**1E-16**
LINE/L2	397294	0.16	**3E-13**	0.22	**1E-16**	0.21	**1E-16**
SINE/Alu	926299	0.10	1E-04	0.22	**1E-16**	0.19	**1E-16**
DNA/hAT-Tip100	26087	0.10	3E-05	0.20	**1E-16**	0.18	**1E-16**
DNA/hAT-Blackjack	17019	0.15	4E-12	0.16	**2E-13**	0.17	**1E-16**
Simple_repeat	343474	0.19	**1E-16**	0.11	**4E-06**	0.16	**8E-14**
LINE/CR1	52244	0.09	4E-04	0.15	**5E-12**	0.13	**9E-09**
DNA/TcMar-Tc2	6979	0.11	1E-05	0.13	**3E-08**	0.13	**3E-08**
DNA/TcMar-Mariner	14046	0.10	5E-05	0.13	**2E-08**	0.12	**2E-07**
CpG	19661	0.06	2E-01	0.10	3E-05	0.11	**1E-06**
LTR/ERVL-MaLR	292520	0.11	**2E-06**	0.09	6E-04	0.11	**8E-06**
LTR/Gypsy?	6912	0.05	1	0.11	**4E-06**	0.08	2E-03
Unknown	6174	0.05	5E-01	0.06	4E-01	0.05	1
LINE/RTE	15421	0.04	1	0.06	2E-01	0.05	1
LTR/Gypsy	9429	0.02	1	0.04	1	0.03	1
DNA/TcMar-Tigger	87328	-0.01	1	0.04	1	0.01	1
LTR/ERVL	134989	-0.03	1	-0.03	1	-0.04	1
LTR/ERV1	139204	-0.13	**7E-09**	-0.10	6E-05	-0.13	**2E-08**
Low_complexity	314872	-0.11	**3E-06**	-0.17	**6E-15**	-0.18	**1E-16**
LTR/ERVK	8019	-0.19	**1E-16**	-0.16	**3E-13**	-0.19	**1E-16**
LINE/L1	767428	-0.16	**8E-13**	-0.19	**1E-16**	-0.20	**1E-16**

1. Repeat classes including more than 6000 repeats were considered for the purpose of analyses.

2. Corr.: correlation coefficients between the recombination sites and specific repeats.

3. P values under the null hypothesis of an absence of correlation. The Bonferroni's correction was applied for multiple comparisons. An adjusted P value > 1 was reported as 1.

4. The values in bold indicate a P value < 1E5

### Relation between recombination sites and the length of tandem repeat sequences

**Figure 6:**
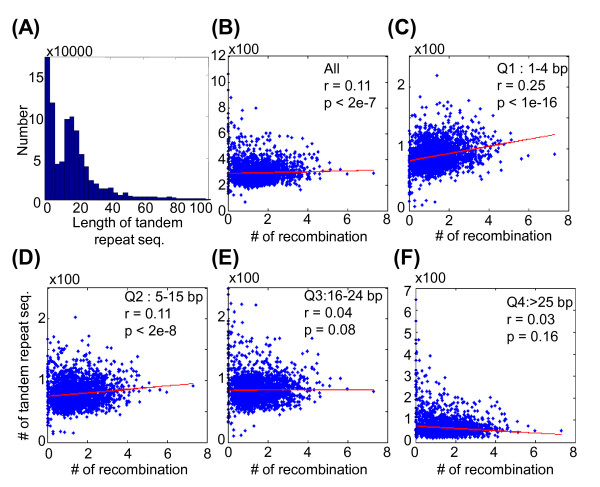
**(A) Distribution of the length of the 947,696 tandem repeats sequences**. (B) Scatter plot of the number of maternal recombination sites and the number of tandem repeat sequences. When the tandem repeat sequences are grouped into 4 quartiles according to the length of repeat sequences, scatter plots for each quartile are shown in **(C) **Q1, 1-4 base pairs (bp), **(D) **Q2, 5-15 bp, **(E) **Q3, 16-24 bp, and **(F) **Q4, larger than 25 bp, respectively. Regression lines are marked in red, and the Pearson correlation coefficients between number of maternal recombination events and the number of tandem repeat sequences are indicated.

Repetitive elements, including tandem repeat sequences, are distributed widely throughout the genome. Tandem DNA repeats are defined as a repeated pattern of two or more nucleotides. The pattern can range in length from 2 to ~100 base pairs (bp) (for example (CATG)n in a genomic region) [26]. In this study, a total 947,696 tandem repeats sequences were identified using the Tandem Repeats Finder [26]. The length distribution of the tandem repeats are shown in Figure 6A, where the 25, 50 and 75 percentile of the length of the tandem repeats were 4, 15 and 24 bp, respectively.

We divided the genome into 2,765 bins of 1-Mb each and determined the number of tandem repeats in each bin. We then analyzed the correlation between the number of maternal meiotic recombination sites and the number of tandem repeats (Figure 6B); the correlation coefficient was 0.11 (P < 2 × 10^-7^). Furthermore, we grouped tandem repeats into 4 quartiles by the length of these repeat sequences, as (Q1) 1-4, (Q2) 5-15, (Q3) 16-24 and (Q4) > 25 bp. The correlation coefficients between recombination sites and the 4 quartiles were 0.25 (P < 1 × 10^-16^), 0.11 (P < 2 × 10^-8^), 0.04 (P = 0.08) and 0.03 (P = 0.16), respectively (Figures 6C-F). These results showed that the maternal meiotic recombination sites were positively correlated with shorter repeat sequences and less correlated with longer repeat sequences. Similarly, we analyzed the correlation between the number of paternal meiotic recombination sites and the number of tandem repeats, with r = 0.12 (P < 5 × 10^-9^). The correlation coefficients for the 4 subgroups were 0.19 (P < 1 × 10^-16^), 0.09 (P < 4 × 10^-6^), 0.09 (P < 3 × 10^-6^) and 0.05 (P = 0.004), respectively (Additional file 4).

## Discussion

In this study, we use a PST approach to analyze the sites of meiotic recombination in two-generation pedigrees. We first tested it on a GMRCL dataset of the Affymetrix SNP 6.0 array consisting of 900 K SNP markers, followed by a 10 K GSE6754 dataset. In the GSE6754 dataset, which was previously used for mapping autism risk loci, most data are based on two-generation pedigrees (1,168 families) as this dataset contains only 29 three-generation pedigrees. Although the PST approach requires only pedigrees of two generations, it requires information from at least two siblings. The use of SNPs as genetic markers to identify recombination sites can often result in the inclusion of uninformative regions. However, the size of uninformative regions that result from the PST approach is significantly lower than that seen from the use of the IBD method (Table 1**)**.

We next assessed whether crossovers may alter the DNA sequence by causing *de novo *mutations at sites of recombination. Given that the uninformative regions of PST were relatively small, eight recombination events were identified with sizes of less than 2 kb. Notably, we did not identify any sequence variation at these recombination points (data not shown). This observation needs further validation by sequencing more datasets.

The average number of recombination events observed with the PST approach was similar to the findings of other studies. The distribution of recombination events showed a mean value of 23.8 in paternal origin and 39.5 in maternal origin. Chowdhury et al reported the genome-wide recombination events in paternal origin ranged from 25.9 to 27.3 while in maternal origin ranged from 38.4 to 47.2 [20]. Another study by Cheung et al demonstrated that the mean numbers of recombination events were 24.0 in male meiosis and 38.4 in female meiosis [15].

In an indirect pedigree analysis using SNPs as genetic markers, Cheung et al [15] reported that several recombination events appeared to occur nearer to the telomeres. Using the PST approach, we analyzed the distance between the recombination site and the centromere for each gender separately (Table 3). In male meiosis, most of the crossovers are located in the q arms, and the number of recombination events increased significantly when moving from centromeres to telomeres. Interestingly, we observed fewer recombination events in the p arms of female chromosomes, resulting in the male-to-female ratio of 1.67 (Table 2). In women, only chromosomes 1q and 6q showed a significant, positive correlation between the number of recombination sites and distance from the centromere (Table 3).

To determine the extensive sequence-context variation in recombination hotspots, Myers et al. constructed a fine-scale map of recombination rates and hotspots across the human genome based on genotypes of 1.6 million SNPs in three sample populations, including 24 European Americans, 23 African Americans, and 24 Han Chinese [27]. The authors reported an increase of recombination hotspots in the regions surrounding coding genes, though these were preferentially located outside the transcribed regions. The analysis of the relationships between recombination hotspots and repeat elements indicated that L2 and THE1B are unusually high in hotspots, whereas L1 elements are low [27]. In this study, we identified a similar pattern of frequent hotspots in L2 as opposed to the low number of hotspots in L1 elements (Table 4**)**. Of note, results showed that the majority of the hotspots in both paternal and maternal meioses were similar.

## Conclusion

Human chromosomes are characterized by prominent differences in the pattern and rate of meiotic recombination events. Significant inter-individual and gender differences also exist. The major advantages of the PST approach include the use of two-generation pedigrees with two or more siblings, fewer uninformative SNP regions, and the ability to perform gender-specific analyses of recombination hotspots (using databases derived from high density arrays such as Affymetrix SNP6.0) and repetitive elements. An accurate determination of meiotic crossovers using this approach may prove useful to explore the biology of human chromosomes.

## Methods

### Identification of meiotic recombination sites

In the present study we compared different SNP-based methods for detecting recombination points, i.e. IBD (Figure 1A) [12], and PST (Figure 1B). The code calling schema for the IBD and PST methods are depicted in the Additional Files 1A and 1B. The meiosis recombination sites were exported from the PSTReader, a MATLAB-based program (version 7.9). The PSTReader was used to define the recombination sites for the IBD and PST methods. The MATLAB source code, example data, and a standalone application can be freely downloaded from: http://www.mcu.edu.tw/department/biotec/en_page/PSTReader/index.htm.

### GMRCL Dataset

In this study, a set of the Affymetrix Genome-Wide Human SNP array 6.0 (GMRCL dataset) consisting of 900 K SNP markers was used as a template. DNA was extracted from blood collected in a study that was approved by the Chang Gung Memorial Hospital Institute Review Board (IRB#99-0229B). SNP genotyping was performed using the SNP array 6.0 (Affymetrix, Santa Clara, CA, http://www.affymetrix.com) at the Genomic Medicine Research Core Laboratory (GMRCL), Chang Gung Memorial Hospital. The GMRCL dataset includes the genotypes of an anonymous family consisting of the paternal/maternal grandfather, paternal/maternal grandmother, father, mother and two children. The identity-delinked SNP genotypes and pedigree information for each member can be downloaded from http://www.mcu.edu.tw/department/biotec/en_page/PSTReader/index.htm.

### GSE6754 Dataset

The GSE6754 dataset was downloaded from the Gene Expression Omnibus (GEO), and contains information from 6,971 Affymetrix GeneChip Human Mapping 10 K 2.0 Arrays. Data from parental and sibling genotypes are available for 1,168 families in this dataset. To increase analytic accuracy, we excluded samples with genotyping call rates less than 90%, those lacking pedigree information, and individuals with chromosomal abnormalities (n = 22) [28]. The remaining 3,864 arrays of 853 families (1,721 parents and 2,145 siblings) were included in the PST analysis of recombination events in human meiosis. The details on individual, families, and pedigrees are provided in Additional file 5.

### Mapping of the recombination sites, repetitive elements and tandem repeat sequences

The recombination sites and repetitive elements were mapped using the hg18 (NCBI Build 36) human reference assembly. The classes and characters of major repetitive elements were downloaded from RepeatMasker [24], and the tandem repeat sequences were identified using the Tandem Repeats Finder program [26]. Correlations between recombination sites and repetitive elements or tandem repeat sequences were analyzed with MATLAB (version 7.9). To assess the distribution and correlation between recombination sites and repetitive elements or tandem repeat sequences, we calculated the number of recombination sites (or repetitive elements or tandem repeat sequences) using a window width set to 1 Mb. We divided the human genome into 2765 bins of 1 Mb each and determined the number of recombination sites in each bin. The distance for each 1 Mb window was calculated based on SNP positions according to the Affymetrix data, assuming a constant crossover rate between two adjacent SNP markers. To calculate the correlation coefficients between the recombination in GSE6754 map, Icelandic map and Marshfield map, we divided the human genome into 2765 bins of 1 Mb each and determined the number of recombination sites in each bin, as described above.

## Abbreviations

PST: parent-sibling tracing; IBD: identity by descent; IBS: identity by state; STRP: simple tandem repeat polymorphisms; SNP: single nucleotide polymorphisms.

## Competing interests

The authors declare that they have no competing interests.

## Authors' contributions

YSL, AC, SMW and THW designed the study and prepared the manuscript. YSL, TC and CHC carried out the statistical analysis. YSL and THW carried out the Affymetrix microarray experiments, obtained the clinical materials and analyzed clinical information. All authors read and approved the final manuscript.

## Supplementary Material

Additional file 1 **Calling schema**. Tables with calling schema for analyzing meiosis, identity by descent (IBD) and parent-sibling tracing (PST). Click here for file

Additional file 2 **Paternal recombination site along the chromosomes**. The paternal recombination site of child 1 and 2 of GMRCL dataset (CH1 and CH2, defined in Figure 1) along chromosomes are demonstrated in figures by the identity by descent (IBD) and parent-sibling tracing (PST) methods. Click here for file

Additional file 3 **Distribution of recombination events**. Figures illustrating the distribution of the 2,145 paternal and 2,145 maternal recombination events in human for each chromosome. Click here for file

Additional file 4 **Correlation between tandem repeats sequences and paternal recombination sites**. Distribution of the length of the tandem repeats sequences and scatter plot of the number of paternal recombination sites with the tandem repeats sequences. Click here for file

Additional file 5 **Detailed information of GSE6754 dataset**. Family ID, individual ID and the pedigree relative of the analyzed 3864 samples which were downloaded from GEO, GSE 6754. Click here for file
