# Supplementary material for: Analysis of human meiotic recombination events with a parent-sibling tracing approach
Source: BMC Genomics. 2011 Aug 26;12:434. doi: 10.1186/1471-2164-12-434 (PMC3186786; doi:10.1186/1471-2164-12-434)
Supplement: Additional file 1 — Calling schema. Tables with calling schema for analyzing meiosis, identity by descent (IBD) and parent-sibling tracing (PST). [file 1471-2164-12-434-S1.DOC]

**Additional File 1A.** Calling schema for identity by descent (IBD) using three-generation pedigree analysis of paternal meiosis #

| **GF** | **GM** | **Father** | **Mother** | **Child** | **Status*** |
| --- | --- | --- | --- | --- | --- |
| AA | AB | AB | AA | AB | GM |
| AA | AB | AB | AB | BB | GM |
| AA | AB | AB | BB | BB | GM |
| AA | BB | AB | AA | AA | GF |
| AA | BB | AB | AA | AB | GM |
| AA | BB | AB | AB | AA | GF |
| AA | BB | AB | AB | BB | GM |
| AA | BB | AB | BB | AB | GF |
| AA | BB | AB | BB | BB | GM |
| AB | AA | AB | AA | AB | GF |
| AB | AA | AB | AB | BB | GF |
| AB | AA | AB | BB | BB | GF |
| AB | BB | AB | AA | AA | GF |
| AB | BB | AB | AB | AA | GF |
| AB | BB | AB | BB | AB | GF |
| BB | AA | AB | AA | AA | GM |
| BB | AA | AB | AA | AB | GF |
| BB | AA | AB | AB | AA | GM |
| BB | AA | AB | AB | BB | GF |
| BB | AA | AB | BB | AB | GM |
| BB | AA | AB | BB | BB | GF |
| BB | AB | AB | AA | AA | GM |
| BB | AB | AB | AB | AA | GM |
| BB | AB | AB | BB | AB | GM |

* GF: grandfather; GM: grandmother

# Situations with no information was omitted.

**Additional File 1B.** Calling schema of the parent sibling tracing (PST) analysis for paternal meiosis.

| **Father** | **Mother** | **Child_1** | **Child_2** | **Status*** |
| --- | --- | --- | --- | --- |
| AB | AA | AA | AA | 1 |
| AB | AA | AA | AB | 0 |
| AB | AA | AB | AB | 1 |
| AB | AA | AB | AA | 0 |
| AB | AB | AA | AA | 1 |
| AB | AB | AA | BB | 0 |
| AB | AB | BB | BB | 1 |
| AB | AB | BB | AA | 0 |
| AB | BB | BB | BB | 1 |
| AB | BB | BB | AB | 0 |
| AB | BB | AB | AB | 1 |
| AB | BB | AB | BB | 0 |

*1: Identical; 0: Non-identical
