# Supplementary material for: Analysis of human meiotic recombination events with a parent-sibling tracing approach
Source: BMC Genomics. 2011 Aug 26;12:434. doi: 10.1186/1471-2164-12-434 (PMC3186786; doi:10.1186/1471-2164-12-434)

**Additional File 2**

The paternal recombination sites of child 1 and 2 derived from the GMRCL dataset (CH1 and CH2, defined in Figure 1) along with each chromosome using the identity by descend (IBD) and parent-sibling tracing (PST) methods. The grandmother and grandfather origin of paternal recombination are indicated as GF and GM, respectively. Both children with identical or not identical origin are indicated with 1 and 0, respectively. The calling schema of IBD and PST methods is shown in **Additional File 1.**


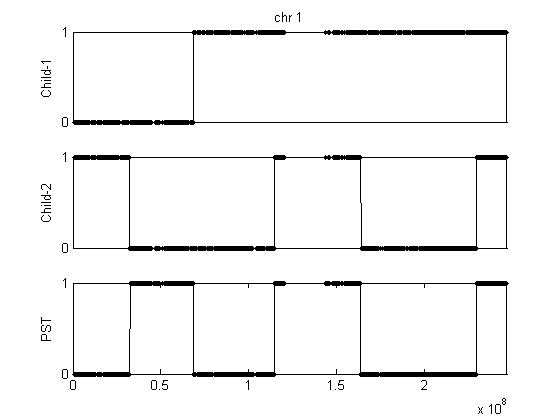

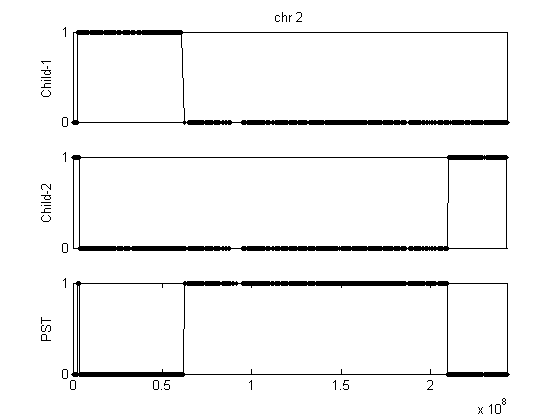

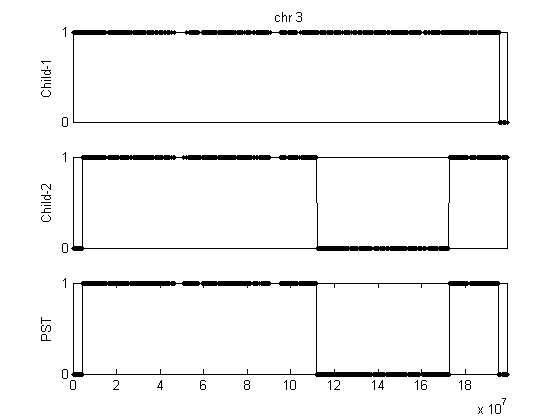

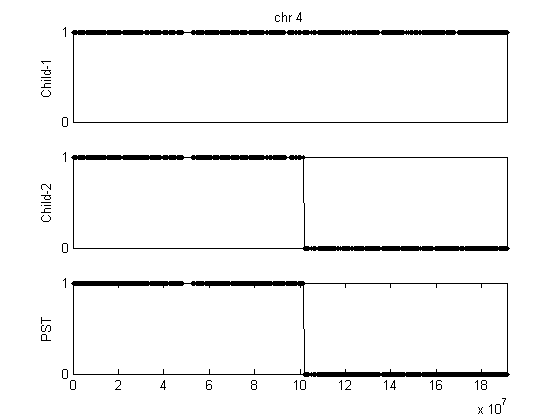

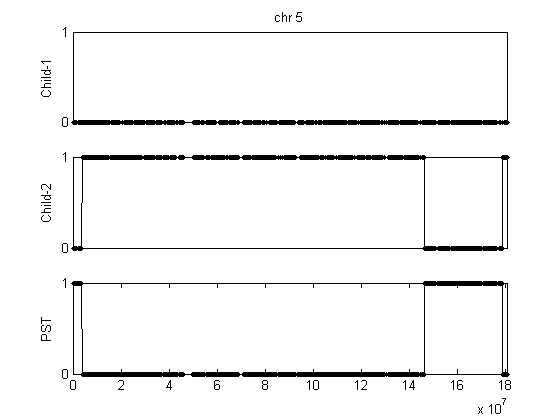

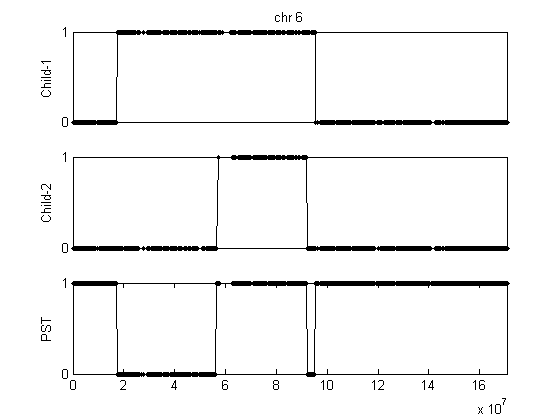

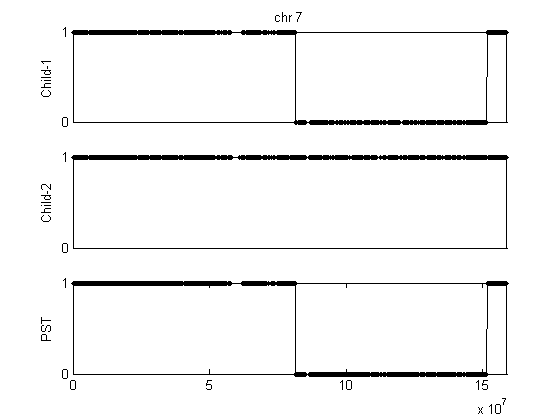

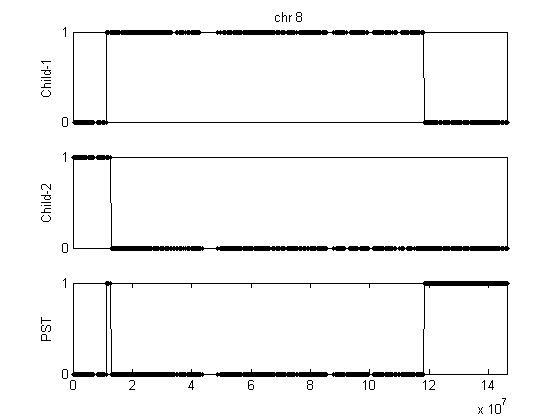

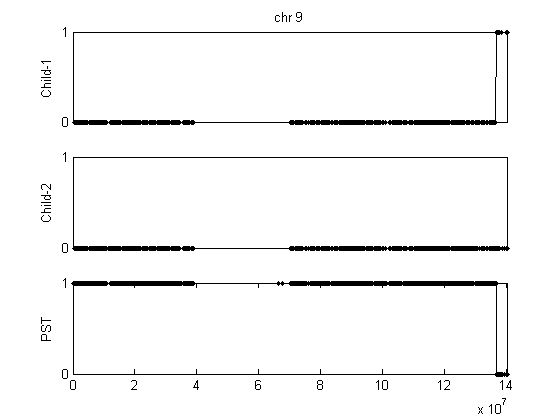


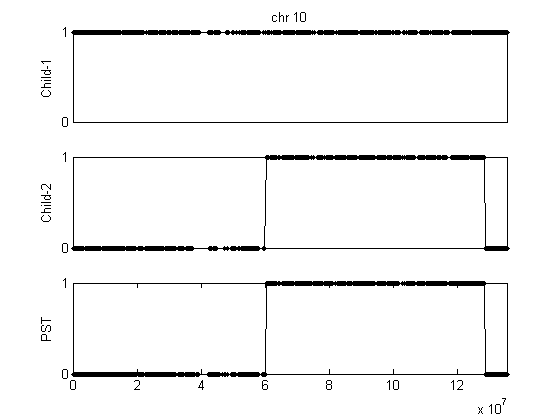

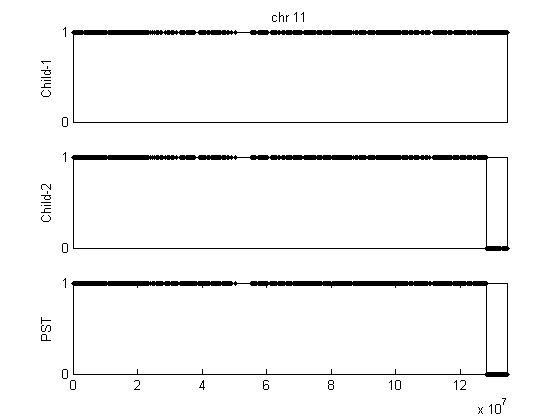

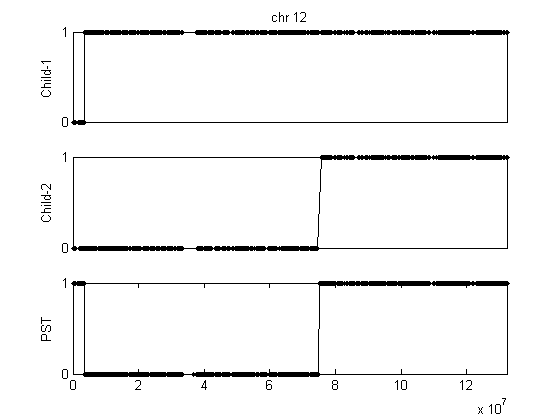

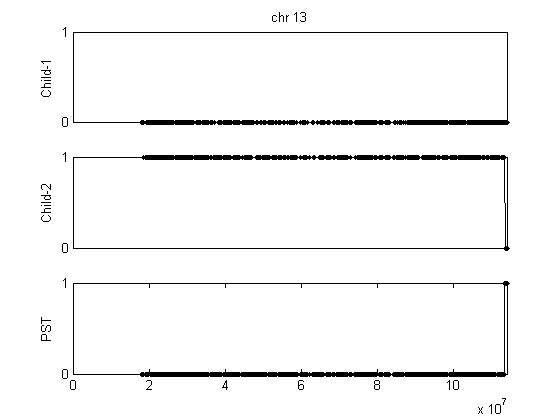

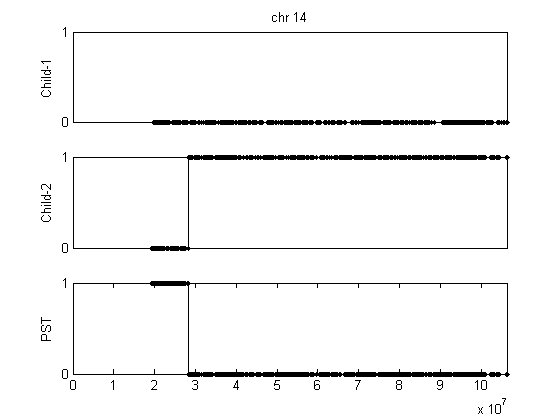

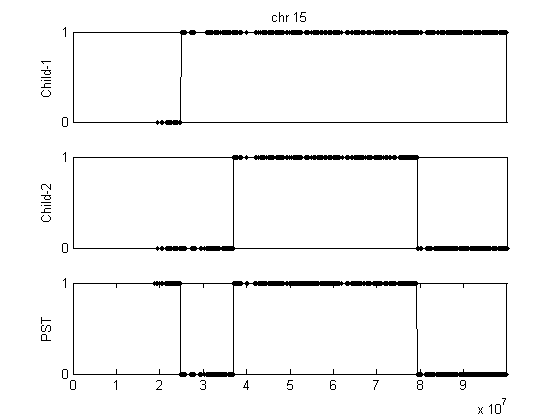

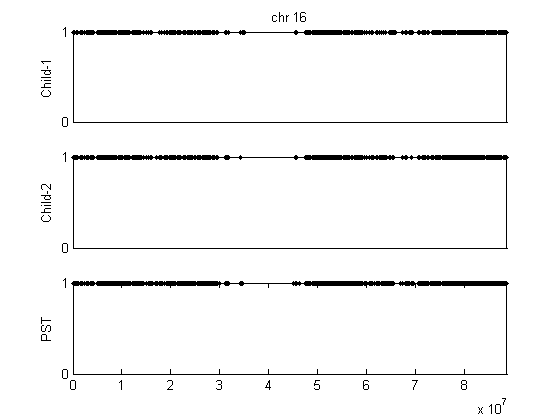

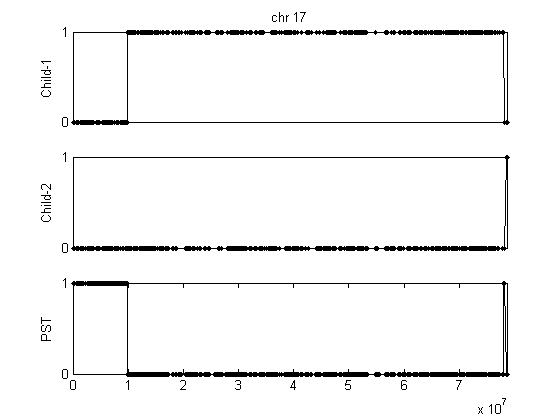

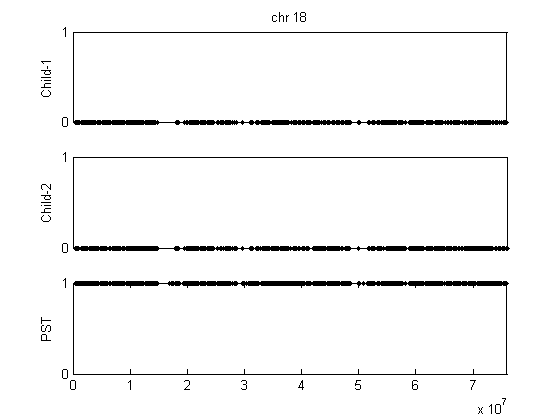

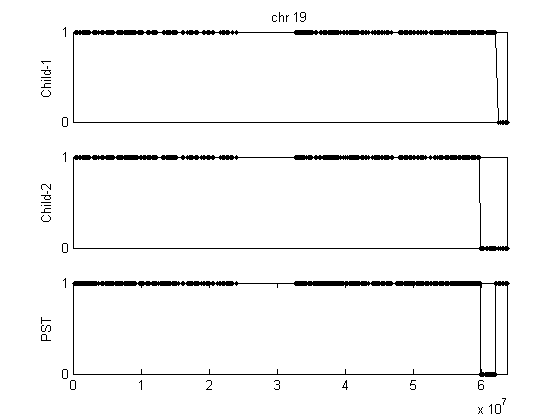

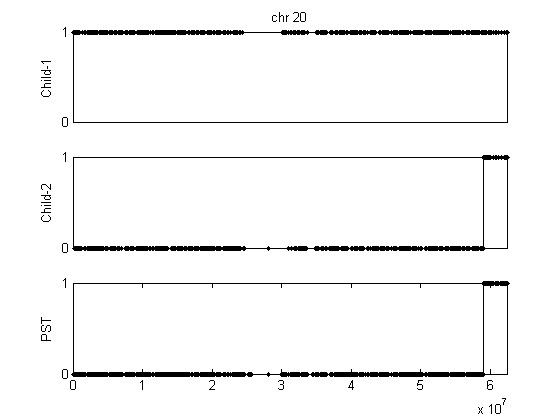

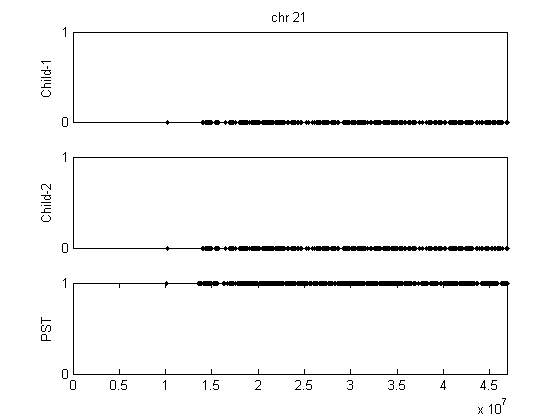

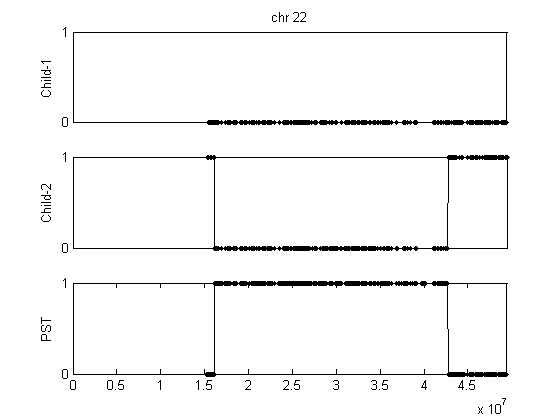

Supplement: Additional file 2 — Paternal recombination site along the chromosomes. The paternal recombination site of child 1 and 2 of GMRCL dataset (CH1 and CH2, defined in Figure 1) along chromosomes are demonstrated in figures by the identity by descent (IBD) and parent-sibling tracing (PST) methods. [file 1471-2164-12-434-S2.DOC]
