# Supplementary material for: Analysis of human meiotic recombination events with a parent-sibling tracing approach
Source: BMC Genomics. 2011 Aug 26;12:434. doi: 10.1186/1471-2164-12-434 (PMC3186786; doi:10.1186/1471-2164-12-434)

**Additional File 3**

The distribution of the 2,145 paternal and 2,145 maternal recombination events in humans for each chromosome. Red and blue lines indicate maternal and paternal crossover rates, respectively. The number of recombination sites was calculated by moving windows 1-Mb in width. The maternal and paternal genetic distance for each 1-Mb window was calculated on the basis of the SNP position information provided by Affymetrix, under the assumption of a constant crossover rate between two adjacent SNP markers. The chromosomal physical position and ideogram are labeled at the top and bottom axes, respectively. The regression lines for maternal (red) and paternal (blue) crossover rates between the distances to centromere are plotted. The chromosome number is indicated at the top of each figure.


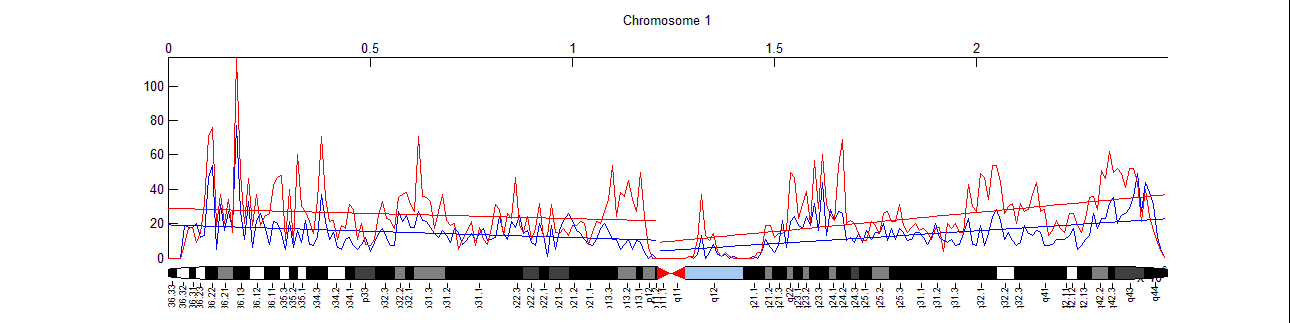


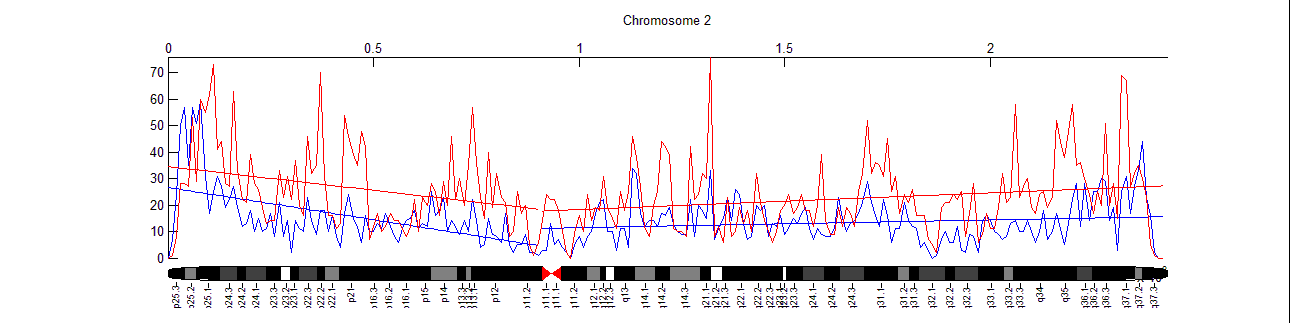

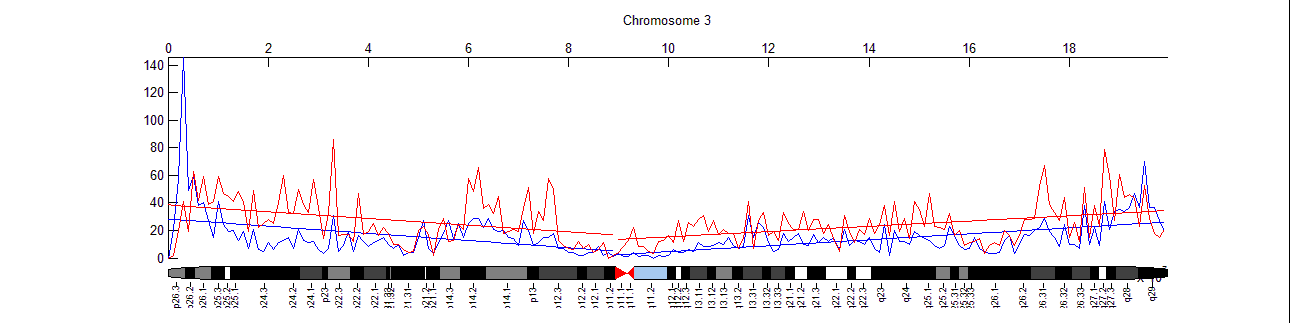

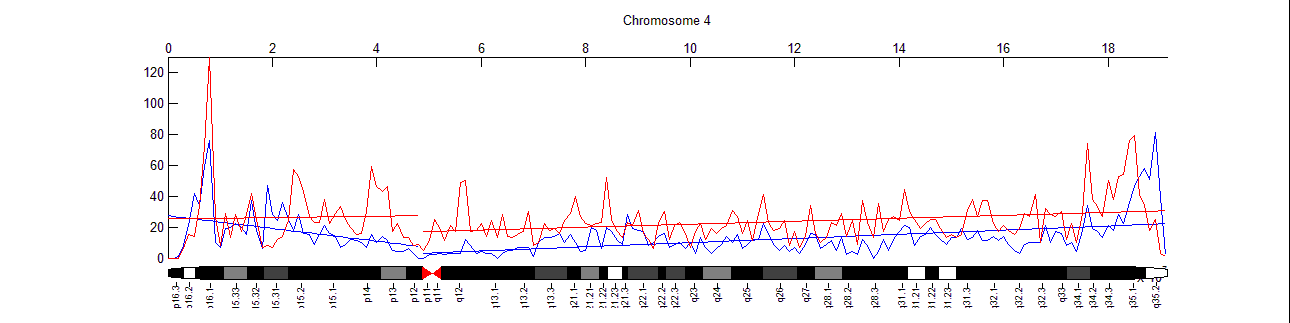

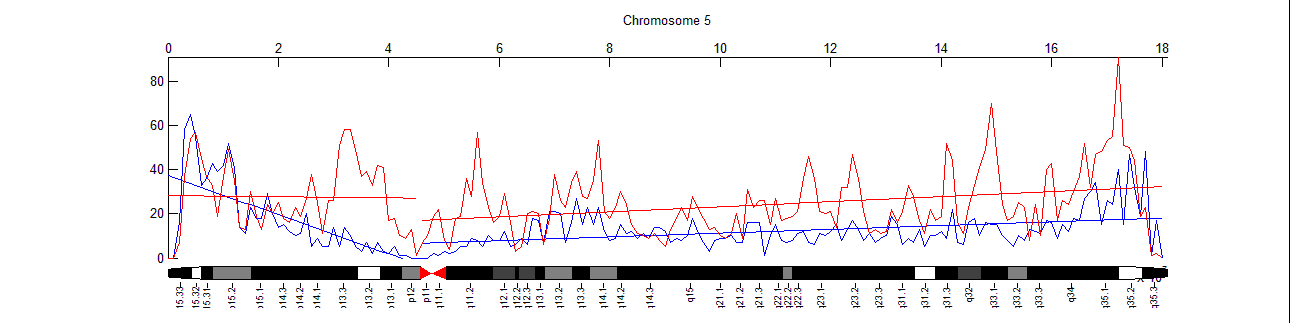

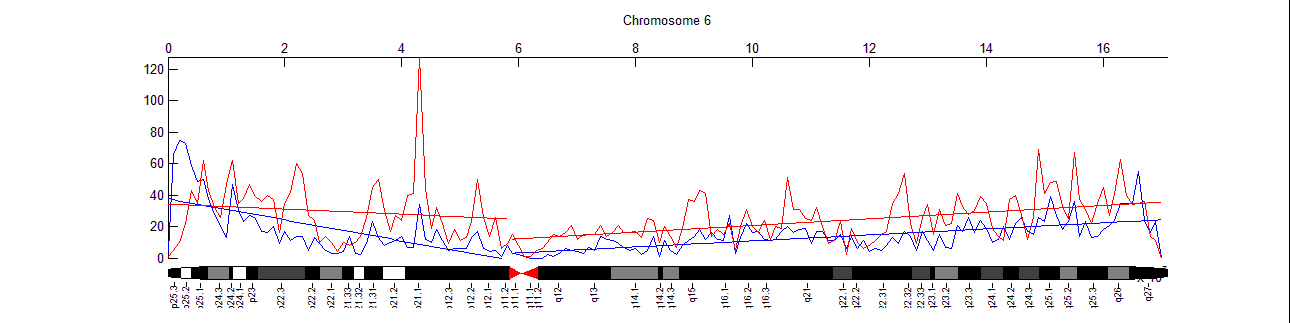

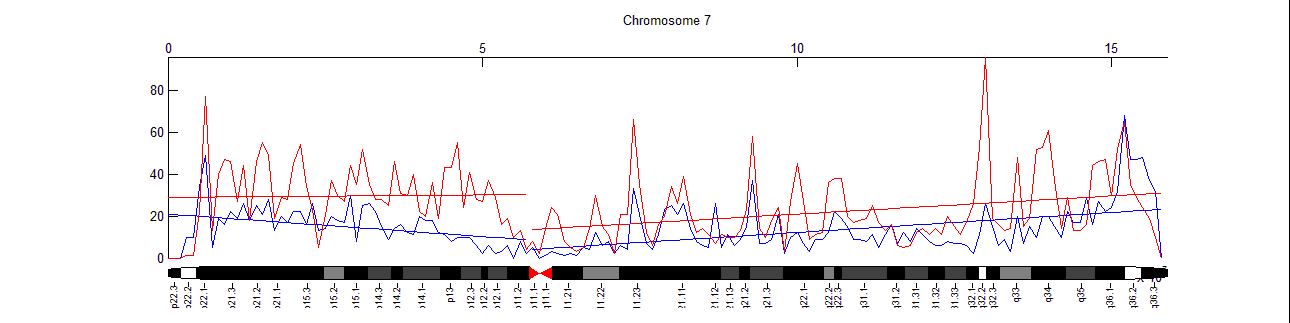

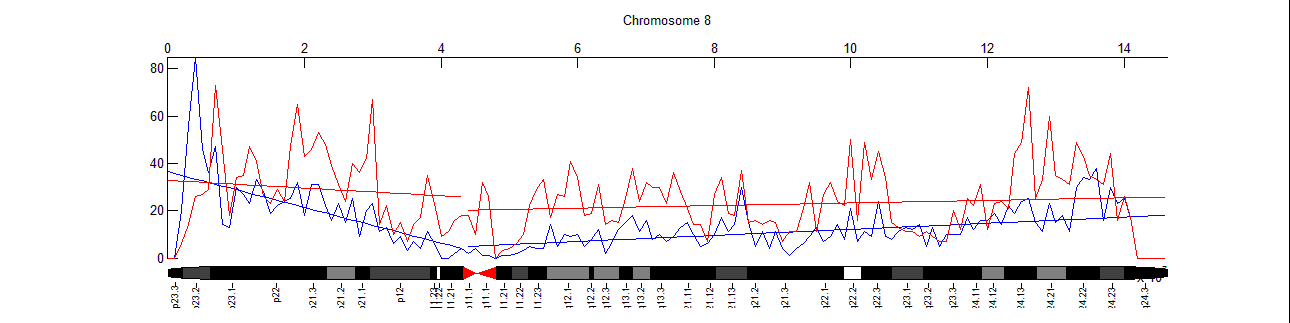

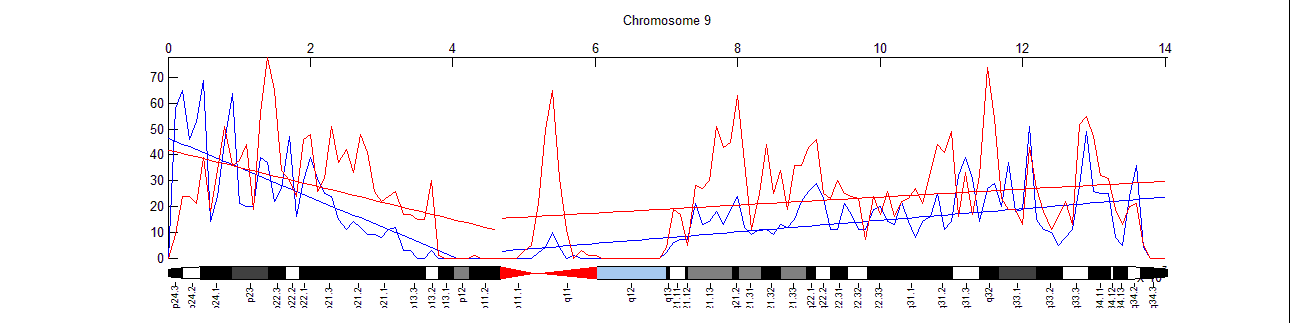

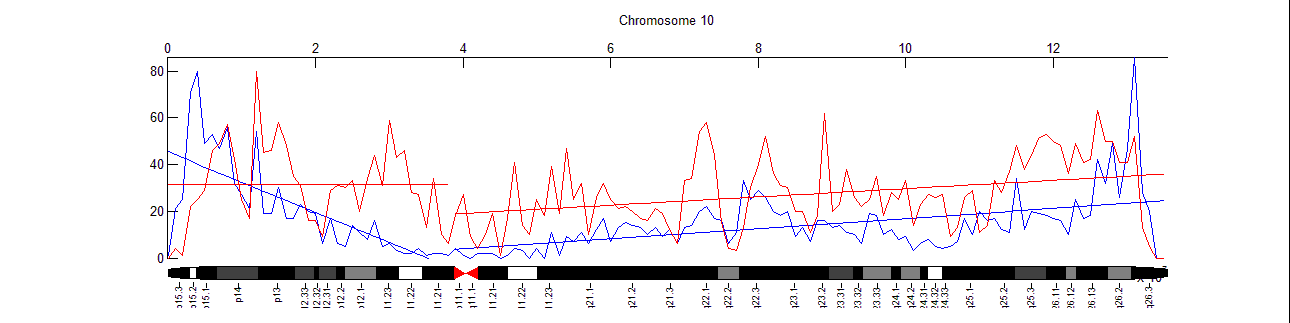

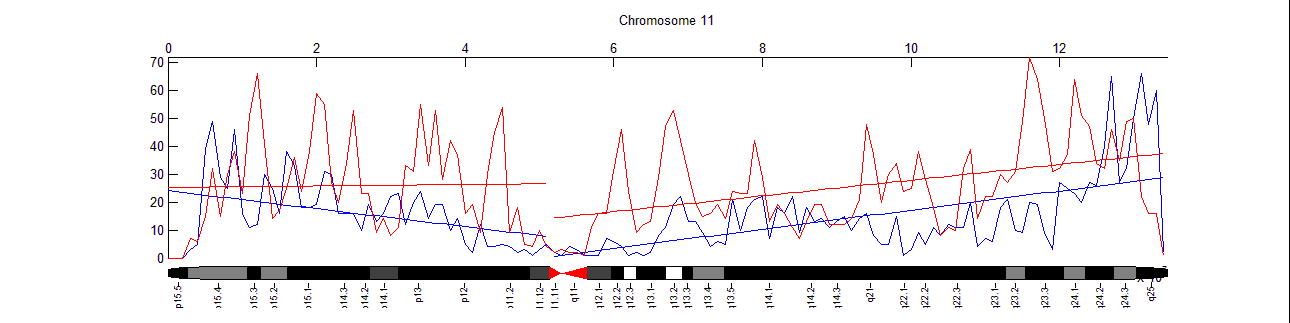

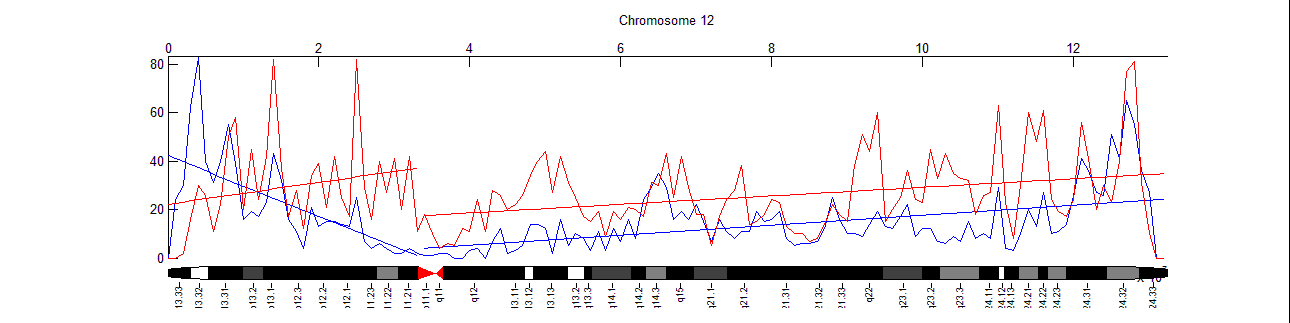

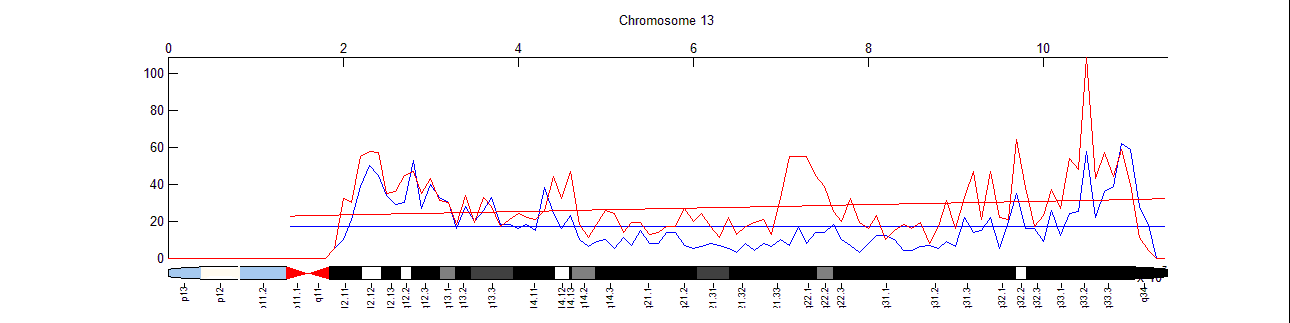

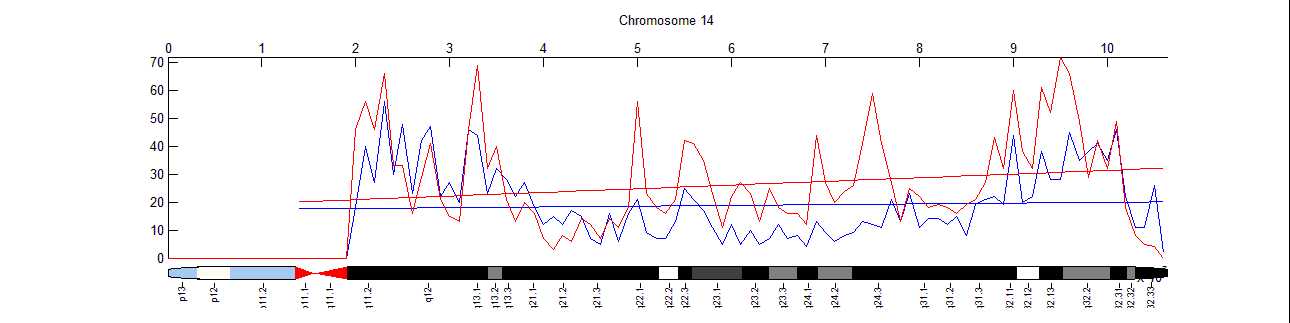

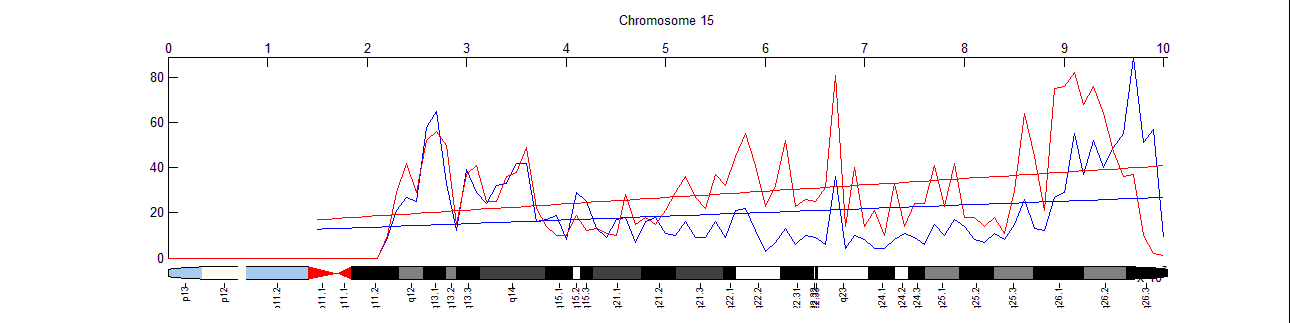

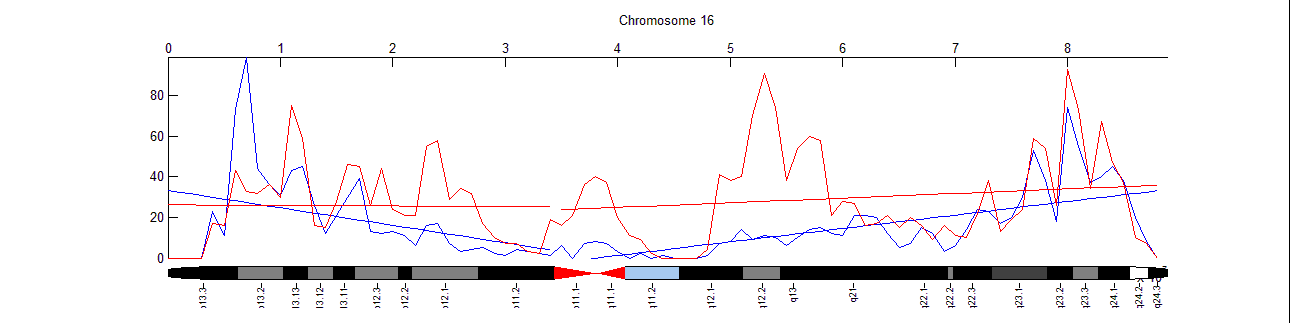

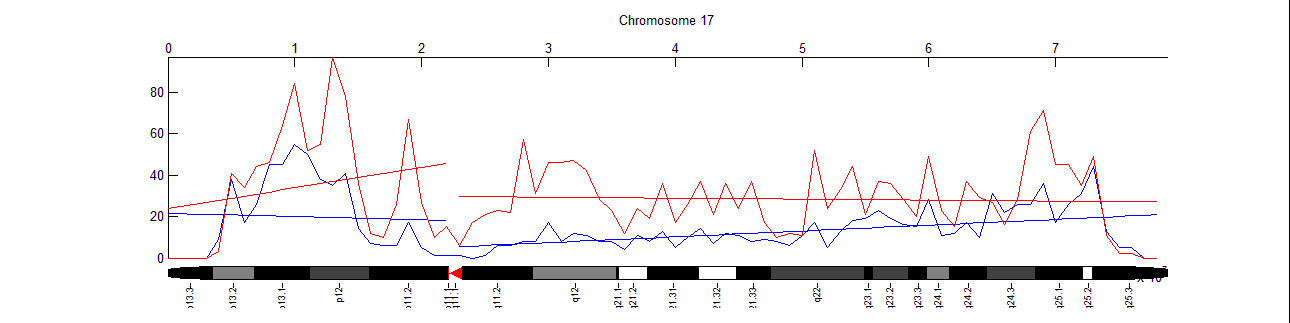

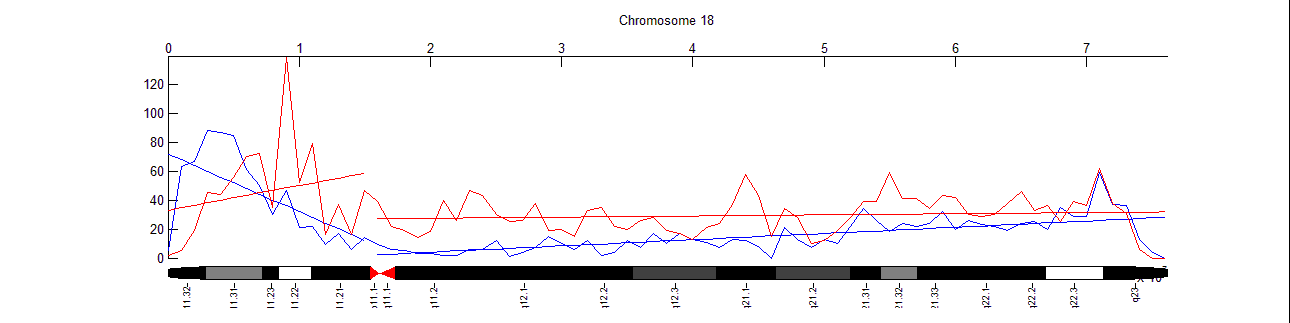

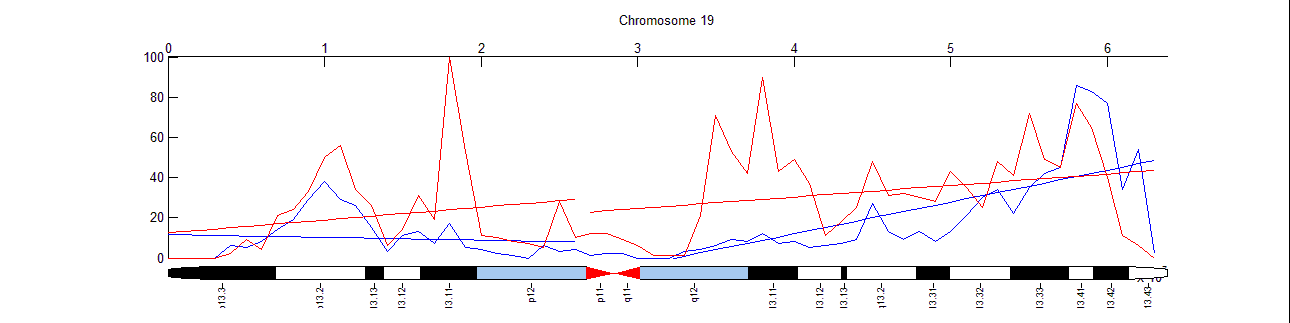

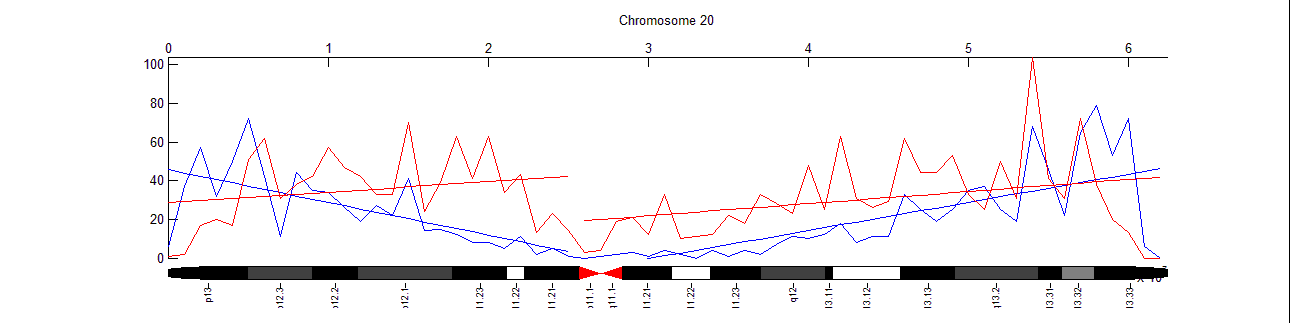

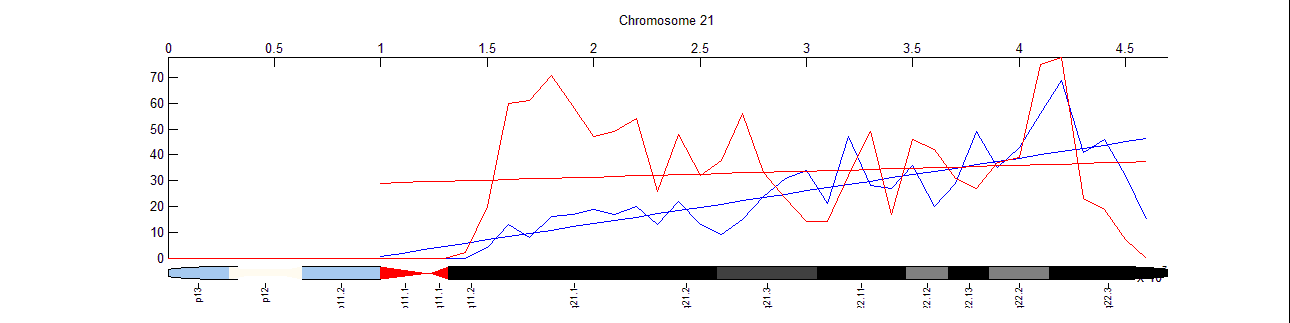

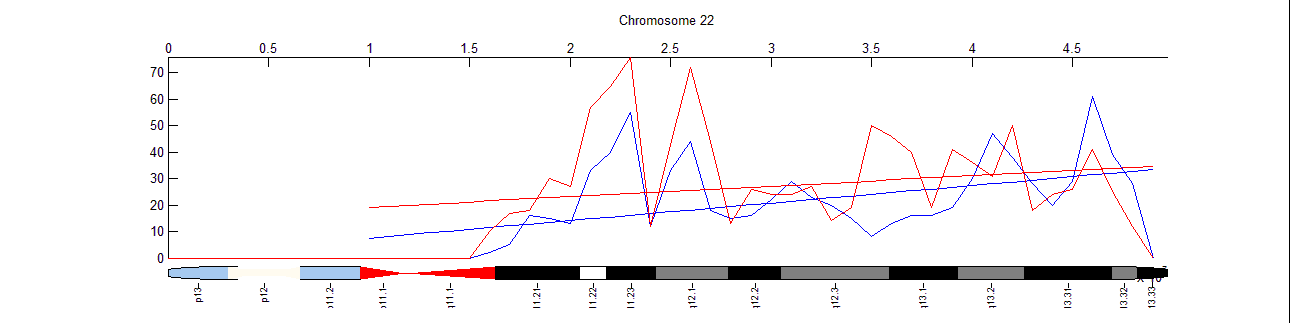

Supplement: Additional file 3 — Distribution of recombination events. Figures illustrating the distribution of the 2,145 paternal and 2,145 maternal recombination events in human for each chromosome. [file 1471-2164-12-434-S3.DOC]
