# Supplementary material for: Analysis of human meiotic recombination events with a parent-sibling tracing approach
Source: BMC Genomics. 2011 Aug 26;12:434. doi: 10.1186/1471-2164-12-434 (PMC3186786; doi:10.1186/1471-2164-12-434)
Supplement: Additional file 4 — Correlation between tandem repeats sequences and paternal recombination sites. Distribution of the length of the tandem repeats sequences and scatter plot of the number of paternal recombination sites with the tandem repeats sequences. [file 1471-2164-12-434-S4.DOC]

**Additional File 4**

**(A) Distribution of the length of the 947,696 tandem repeats sequences. (B) Scatter plot of the number of paternal recombination sites with the number of tandem repeat sequences.** When the tandem repeat sequences are grouped into 4 quartiles according to the length of repeat sequences, scatter plots for each quartile are shown in **(C)** Q1, 1-4 base pairs (bp), **(D)** Q2, 5-15 bp, **(E)** Q3, 16-24 bp, and **(F)** Q4, larger than 25 bp, respectively. Regression lines are marked in red, and the Pearson correlation coefficients between number of maternal recombination sites and the number of tandem repeats sequences are indicated.
